# Conceptual Structure within and between Modalities

**DOI:** 10.3389/fnhum.2012.00333

**Published:** 2013-01-02

**Authors:** Katia Dilkina, Matthew A. Lambon Ralph

**Affiliations:** ^1^Neuroscience and Aphasia Research Unit, School of Psychological Sciences, University of ManchesterManchester, UK

**Keywords:** concepts, conceptual organization, semantic system, modality-specific knowledge, multimodal knowledge, hub-and-spoke model

## Abstract

Current views of semantic memory share the assumption that conceptual representations are based on multimodal experience, which activates distinct modality-specific brain regions. This proposition is widely accepted, yet little is known about how each modality contributes to conceptual knowledge and how the structure of this contribution varies across these multiple information sources. We used verbal feature lists, features from drawings, and verbal co-occurrence statistics from latent semantic analysis to examine the informational structure in four domains of knowledge: perceptual, functional, encyclopedic, and verbal. The goals of the analysis were three-fold: (1) to assess the structure within individual modalities; (2) to compare structures between modalities; and (3) to assess the degree to which concepts organize categorically or randomly. Our results indicated significant and unique structure in all four modalities: perceptually, concepts organize based on prominent features such as shape, size, color, and parts; functionally, they group based on use and interaction; encyclopedically, they arrange based on commonality in location or behavior; and verbally, they group associatively or relationally. Visual/perceptual knowledge gives rise to the strongest hierarchical organization and is closest to classic taxonomic structure. Information is organized somewhat similarly in the perceptual and encyclopedic domains, which differs significantly from the structure in the functional and verbal domains. Notably, the verbal modality has the most unique organization, which is not at all categorical but also not random. The idiosyncrasy and complexity of conceptual structure across modalities raise the question of how all of these modality-specific experiences are fused together into coherent, multifaceted yet unified concepts. Accordingly, both methodological and theoretical implications of the present findings are discussed.

## Introduction

We experience objects and entities in the world through many different modalities. We experience them perceptually through observation (visual, auditory, olfactory, gustatory, and tactile), as well as functionally through interaction and use. Remember the eggs that you had for breakfast this morning? Consider the richness of this simple perceptual and motor experience. In addition, we experience objects contextually or in relation to other objects, entities, or places. For example, eggs are usually eaten at breakfast, often accompanied by sausage or bacon. Finally, there is the abundant verbal experience when we read, write, or talk about things in the world.

Each of these modalities provides a rich and unique experience, and contributes to our semantic knowledge – our cross-modal conceptual knowledge. All contemporary theories of semantic memory and its neural basis share the assumption that semantic representations are formed from multimodal experience, coded in distinct modality-specific brain regions. Furthermore, the regions representing information relevant to a specific item are activated during semantic processing whether or not this type of information is explicitly required by the task/activity. Considerable evidence has been accumulated in favor of these ideas (see Martin, 2007; Patterson et al., 2007; Thompson-Schill, 2003 for reviews). For example, in a PET neuroimaging paradigm, Martin et al. (1995) asked a group of participants to name either the color or the action associated with visually presented objects. They found that generating action words activated the left posterior middle temporal gyrus (associated with visual motion processing), while producing color words activated the fusiform gyrus (associated with visual form and color processing). Similar patterns of activation were obtained in different experimental paradigms which did not explicitly present or require object properties of specific knowledge type, including brief picture viewing (Chao et al., 1999), picture naming (e.g., Martin et al., 1996; Chao et al., 1999; Moore and Price, 1999), visual match-to-sample (Chao et al., 1999), and same/different judgments with pairs of pictures or words (Perani et al., 1999). In all of these studies, manipulable objects such as tools tend to activate the left posterior MTG more than living things such as animals, which preferentially engage posterior inferior temporo-occipital regions. More detailed investigations have identified distinct areas in the left posterior lateral temporal lobe responding to biological motion vs. object motion (e.g., Beauchamp et al., 2002). The distributed nature of conceptual representations has been investigated not only in the visual and motor modalities, but also in other perceptual modalities. For example, Simmons et al. (2005) presented their participants with pictures of appetizing foods along with pictures of locations. They used a simple same/different judgment task to elicit fMRI activation contrasts for these two categories. The results showed that even in this simple task, food items preferentially activated two areas associated with gustatory/olfactory processing – the right insula and the left orbitofrontal cortex (see also Goldberg et al., 2006; González et al., 2006). These neuroimaging findings have been complemented by neuropsychological reports of patients with category-specific semantic deficits (greater impairment for living vs. non-living things or vice versa, greater impairment for fruits and vegetables vs. tools, and so on), where lesion locations match the brain areas and functional specialization suggested by the functional imaging studies with unimpaired individuals (e.g., Tranel et al., 1997).

Another group of related studies have explored the relationship between conceptual categories and different types of modality-specific information sources by analyzing verbally generated feature lists from unimpaired individuals. For example, Garrard et al. (2001) collected directed feature norms for 62 items from six categories. They classified the features as sensory, functional, or encyclopedic (see Materials and Methods) and investigated how the three types of features compared across the six categories. They found that living things tended to have more sensory than functional attributes compared to non-living things; they also had more encyclopedic attributes. Furthermore, living things had less distinctive and more shared features than non-living things. A similar investigation, though on a substantially larger scale, was conducted by Cree and McRae (2003), who collected verbal feature norms for 541 concrete concepts. The authors argued that a full understanding of category-specific deficits would have to go beyond the distinction between sensory and functional features. They proposed a classification consisting of nine different knowledge types processed in distinct neural regions (Table 1). Using their detailed classification, Cree and McRae (2003) showed that all feature types play a role in distinguishing among conceptual categories, though admittedly some knowledge types were more relevant to specific categories than others. For example, not surprisingly, the feature type of visual motion was important for the category of creatures but unimportant for fruits and vegetables, which relied more on visual-color features, etc. The results from a large hierarchical cluster analysis combining the knowledge types with a number of other factors including the proportion of distinguishing features for each category, visual similarity and complexity, semantic similarity, concept familiarity, and word frequency, demonstrated a unique and significant contribution of all factors to the conceptual structure present in their data set. In order to go beyond just those feature types that are most readily reported verbally, Hoffman and Lambon Ralph (2012) asked participants to rate the importance of each sensory and verbal modality to 100 different concepts. Not only did the results complement the previous verbal feature listings but the study found that information arising in other modalities such as sound, motion, smell, and taste (which are rarely reported in verbal listing studies) also provided important differential experience across categories.

**Table 1:** **Types of knowledge and associated brain regions assumed by Cree and McRae (2003)**.

Knowledge type	Brain region
Visual – color	Bilateral posterior ventral temporal cortex
Visual – parts and surface properties	Bilateral ventral occipital cortex
Visual – motion	Left posterior middle temporal gyri
Tactile	Dominant hand and finger areas of primary somatosensory and motor cortices
Olfactory	Piriform cortex and right lateral orbitofrontal cortex
Gustatory	(Left) anterior insula and orbitofrontal and precentral gyri^†^ (Kobayashi, 2006)
Auditory	Bilateral posterior superior temporal gyrus^†^
Functional	Left ventral premotor cortex
Encyclopedic	Multiple regions

*^†^Area not specified by Cree and McRae (2003)*.

In summary, previous investigations have focused on the relationship between categories and feature types. While these studies have provided important insights about semantic representation and the nature of category-specific deficits, in this investigation we returned to consider the primary hypothesis held by contemporary theories of semantic memory – namely that concepts arise from the convergence of our multimodal, verbal, and non-verbal experience. As such, it becomes important to understand the distribution and structure of representation in each modality and how this contributes to the overall multimodal semantic representation. Accordingly, a series of fundamental questions arise: What does the structure within each of these modalities look like? Is it random? Is it categorical/taxonomic? What principles govern the organization of information within modality? How do they compare across modalities?

The goal of this study was to investigate the formation of concepts overall. Thus, we took a novel approach in order to look in more detail at four different modalities of knowledge – visual, verbal, encyclopedic, and functional. We investigated how each modality contributes to the overall semantic representation and how the structure in each modality varies.

There are different ways in which each modality can be probed empirically and how the data arising are treated. We, therefore, compared across the methods directly. As far as we are aware, these direct comparisons have not been made before. For example, we compared information about visual experience as derived from verbal feature listings (two methods each deriving features in a slightly different way) as well as those extracted from participants’ drawings of the same concepts. Secondly, we compared feature data (itself a reflection of verbal experience) against the structure present in a very large verbal corpus which does not attempt to derive attributes but instead utilizes co-occurrence statistics to infer the underlying representations [in this case, derived from latent semantic analysis (LSA) of the British National Corpus].

Our study had three distinct goals: (1) to establish the organization of information arising in each modality of knowledge; (2) to compare the structure between the various modalities; and (3) to assess the degree to which concepts in each modality are organized taxonomically or randomly.

## Materials and Methods

We utilized four different data sets reported in the literature, which gave us four different types of representations – perceptual, functional, encyclopedic, and verbal. Each of the data sets is described below.

### Garrard et al. feature listings data set

Garrard et al. (2001) asked 20 adult volunteers (mean age 67 years old) to list features for each of 62 items (originally 64 but two of the items were subsequently excluded). The participants were prompted to list the category of each item as well as two to six descriptive features (“an elephant is…”), two to six parts features (“an elephant has…”), and two to six abilities-and-uses features (“an elephant can…”). After the initial data collection, the features were processed to use standardized wording (since a given feature can often be described in multiple ways) and to exclude qualifying, exemplifying, or highly idiosyncratic information. Only features listed by at least two participants were considered. They were classified as sensory, functional (action, activity, or use of an item), encyclopedic (associative relationships), or categorical. The set of features consisted of 50% sensory, 28% functional, 15% encyclopedic, and 7% categorical. We used the sensory, functional, and encyclopedic features from this set.

### Cree and McRae feature listings data set

Cree and McRae (2003) reported a list of features produced by undergraduate students for a set of 541 concepts commonly used in categorization and semantic memory tasks. In their feature elicitation method, each participant was presented with 20 or 24 (mostly dissimilar) concept names alongside 10 blank lines to be filled with features of each item. Thirty participants provided features for each of the 541 concepts. Only features that were listed by at least five participants were included in the report. The feature listings were not edited in any way. Excluding taxonomic labels, the authors classified each of the features as belonging to one of nine types: seven perceptual types (visual – color, visual – parts, and surface properties, visual – motion, smell, taste, touch, and sound), a functional type (how one interacts with the item), and an encyclopedic type (all non-perceptual and non-functional descriptors). They reasoned that these were widely accepted knowledge types, which are processed in distinct neural regions (see Table 1).

Since most of the perceptual knowledge types included few features, we combined them into a single perceptual classification, along with the functional and encyclopedic feature types – a classification comparable to the one used by Garrard et al. (2001).

### Rogers et al. picture drawings data set

Instead of using verbal feature listings, Rogers et al. (2004) asked eight participants (mean age of 62) to draw from name the same 64 items as Garrard et al. (2001) had presented to their participants for verbal feature generation. The subjects had 1 min to draw each item and were told that their drawings would not be judged for artistic merit but be assessed for the degree to which they correctly represented the nature of the object. Two independent raters compiled lists of all the visual features present each drawing. Features included by only a single participant were excluded. After this initial data collection, the feature lists were compared to the drawings once more and features that described overlapping or similar aspects of the drawings were combined together to produce the final visual feature description of each item. We considered the Rogers et al. set as another source of information about how perceptual experience contributes to our conceptual knowledge and compared this information source to the subset of verbal features given a perceptual classification (see above).

### Hoffman et al. latent semantic analysis data set

The final data set we utilized did not include feature lists. Instead, it provided representations for numerous items based on their patterns of occurrence in verbal context. Hoffman et al. (2011, 2012) performed LSA on the British National Corpus, which contains over 87 million words in 3125 documents. The authors split the original documents into 1000-word-long samples, which gave them 87,375 smaller documents. Only words that appeared at least 50 times in the corpus and in at least 40 different documents were included. The resultant LSA produced 300-dimensional representational vectors for 38,456 words^1^.

In order to compare the representations across the various knowledge types, we took the intersection of these data sets, which gave us a list of 52 concepts. Table 2 presents basic statistics for each of the feature data sets. Two things should be noted. First, even though the 52 items were present in each data set, they did not necessarily have entries for all feature types: 47 items had encyclopedic features in the Garrard et al. set; another subset of 47 had encyclopedic features in the Cree and McRae set; and a smaller subset of 38 had functional features in the Cree and McRae set. Secondly, an important aspect of the feature-based representations is their density. While all three data sets included a great number of features, the percent of features per item, that is the average number of features listed for a single concept divided by the total number of features of this type (i.e., representational density), was relatively low. Most strikingly, the Cree and McRae data set, which was the only one where the initially collected lists of features were not further processed or edited, had the lowest density, for all feature types.

**Table 2:** **Descriptive statistics for each of the data sets**.

Data set	*N* concepts	*N* features	Average *N* concepts per feature	Average *N* features per concept	Density of representation (% feats/concept)
Rogers et al. visual	52	194	3.89	14.50	7.5
Garrard et al. sensory	52	206	3.00	11.87	5.8
Garrard et al. functional	52	144	2.27	6.29	4.4
Garrard et al. encyclopedic	47	79	2.23	3.74	4.7
Cree and McRae perceptual	52	208	2.09	8.35	4.0
Cree and McRae functional	38	87	1.32	3.03	3.5
Cree and McRae encyclopedic	47	112	1.39	3.21	3.0
Cree and McRae edited perceptual	52	214	4.22	17.37	8.1
Cree and McRae edited functional	52	81	2.68	4.17	5.2
Cree and McRae edited encyclopedic	52	115	2.86	6.33	5.5

Why might low representational density indicate a problem? When there is a great number of features but each of these features applies to singular or few items, it is possible that there is some information missing − that some of these features in fact apply to more of the items. The problem is more severe than simply that of “missing” information because, in a binary type of representation, each item either has a feature or it does not; there is no such thing as “unknown.” So when a feature is missing, it is effectively non-existent for the item. For example, when the data set fails to specify that a dog has a neck, it in fact specifies that a dog does not have a neck.

Since we had multiple sets providing overlapping categories of features, it became clear that one set (Cree and McRae) repeatedly exhibited lower representational density compared to the corresponding sets from alternative sources. To ensure that the low representational density of this data set did not indicate the problem described above, we edited the feature list for each concept in a fashion similar to Rogers et al. (2004) Specifically, we checked all possible features against each of the 52 concepts and wherever a feature was judged to be true of an item but was not marked in the data set, it was added. Labels that described the same feature were combined together as per Garrard et al. (2001). Finally, a few extra features were added to ensure that each item had at least one feature in each knowledge type. The statistics for the edited sets are also shown in Table 2. After these edits, the representational density of the Cree and McRae data set increased substantially and was now comparable to that of the other sets.

Finally, it should be noted that in both the Garrard et al. and the Cree and McRae data sets (including the edited version), the sensory/perceptual representations were denser than both the functional and the encyclopedic representations. Since this was true of both feature lists, it is very likely that it is true of people’s mental modality-specific representations as well. Unfortunately, the nature of the LSA vectors did not allow us to compute a similar measure for verbal representations.

## Analysis and Results

### Conceptual structure within individual modalities

Our first goal was to assess the representational structure that each modality gives rise to. We took three distinct approaches: (1) hierarchical cluster analyses, using the Euclidean distances between pairs of items (the feature-based vectors of each concept) to build dendrograms depicting the structure in each data set; (2) correlational analyses, computing correlations between pairs of items in each data set, giving rise to correlational plots depicting the similarity structure (as opposed to distance or dissimilarity); and (3) a different computation of the similarity between concepts, this time using the cosine between pairs of vectors – for each item in each data set, we produced a list of most similar concepts. All analyses reported in this and following sections were computed in the statistical package R using standard parameter settings. The results are reported grouped by knowledge type/modality.

#### Sensory/perceptual representations

**Figure 1:**
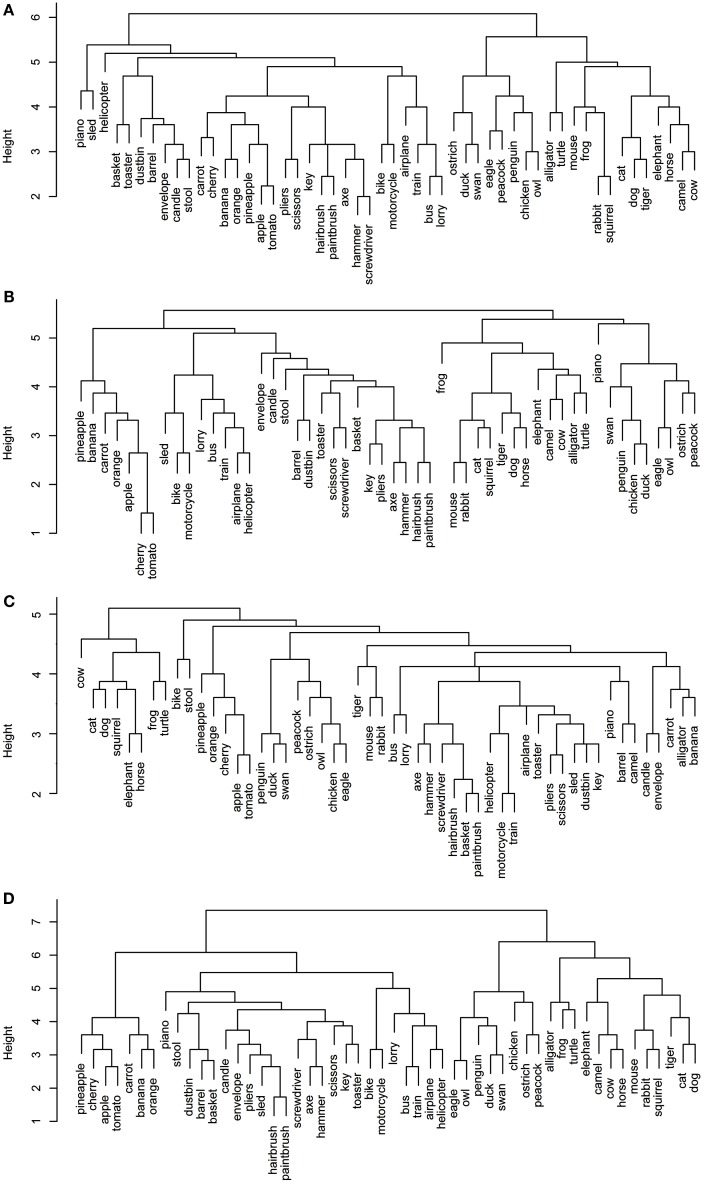
**Dendrograms from hierarchical cluster analysis for each sensory/perceptual data set: (A) Rogers et al. visual; (B) Garrard et al. sensory; (C) Cree and McRae perceptual; (D) Cree and McRae edited perceptual**.

**Figure 2:**
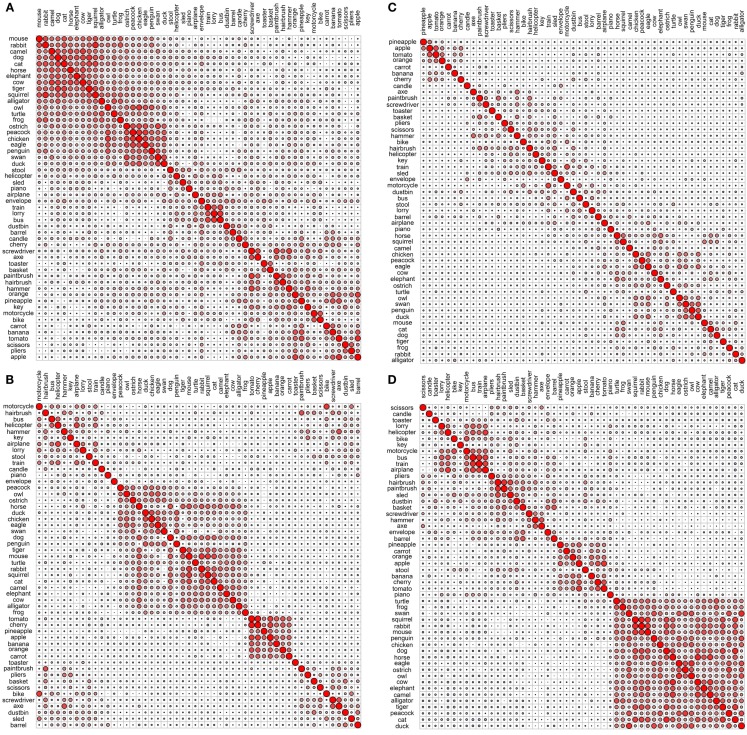
**Correlational plots for each sensory/perceptual data set: (A) Rogers et al. visual; (B) Garrard et al. sensory; (C) Cree and McRae perceptual; (D) Cree and McRae edited perceptual**. The relative size of the circles represents the relative magnitude of the corresponding Pearson correlation coefficient; positive correlations are in red, negative are in blue. The ordering of the concepts along the two axes is identical and it is generated automatically to best depict clusters of concepts with high inter-correlations. As a result, the ordering may differ between graphs.

Figures 1 and 2 depict the plots for the four data sets within the sensory/perceptual modality. They all show a relatively detailed structure, which generally follows categorical distinctions. For example, there is a basic separation between the animals and the non-animals. Furthermore, the birds form a subcategory within the animal group. Within the non-animal group, the fruits and vegetables tend to cluster together, as do the vehicles.

Each data set comes with its own curious idiosyncrasies and interesting trends. For example, in the Rogers et al. visual set (Figures 1A and 2A), the animal category forms sensible subcategories, including large land animals (*elephant*, *horse*, *camel*, and *cow*), small land animals (*rabbit*, *squirrel*, *frog*, and *mouse*), canine and felines, and reptiles. Amongst the cluster of birds, *chicken* is grouped with *owl*, and then *penguin* in the dendrogram, but the correlational plot shows that *chicken* is in fact most highly correlated with *peacock*. Within the non-animal category, in addition to the fruits and vegetables and the vehicles, there is also a large cluster of implements with coherent taxonomic substructure (*hammer* and *screwdriver*, *pliers* and *scissors*, *hairbrush* and *paintbrush*). Notably, two of the vehicles, *helicopter* and *sled*, do not group with the rest from their category in the dendrogram, but the correlational plot again shows inter-correlations among the whole class. Finally, the correlational graph reveals that the associations among the non-animals are much weaker than those among the animals.

In the Garrard et al. sensory feature dendrogram (Figure 1B), with the exception of the bird subcategory, the animals do not organize into subclusters as neatly as they did in the Rogers et al. set. Also, *piano* curiously clusters with the bird group instead of the non-animals (though inspection of the correlational plot proves that this item correlates most with a few of the artifacts, and even those correlations are very weak). Within the non-animals, the fruits and vegetables all group together, as do the vehicles (which form two subclusters – smaller vs. larger vehicles). While certain tools pair together as seen before (e.g., *hairbrush* and *paintbrush*), there is no coherent cluster of implements. Figure 2B shows that the artifacts correlate with each other, but considerably more weakly than the living things (as seen previously). In this set, the category of fruits and vegetables appears much more distinct.

It is likely that many of the differences between the organization seen in the Rogers et al. set vs. the Garrard et al. as well as the Cree and McRae sets stem from the fact that the latter include not only static (colorless) visual information but also other perceptual features. It is also possible that drawings provide a more direct sample of true visual experience whereas feature elicitation is inevitably somewhat influenced by the demands and vocabulary-availability of speech production (see Rogers et al., 2004; Hoffman et al., 2011; Hoffman and Lambon Ralph, 2012, for further discussion of this issue).

The original Cree and McRae perceptual set exhibits the most puzzling structure. As can be seen in Figure 1C, the birds form their own cluster but they group with the artifacts instead of the other animals. The set of animals itself does not group entirely together – *tiger*, *mouse*, and *rabbit* form their own little cluster, which joins with the artifacts; and *camel* clusters with *barrel* and *piano*. The vehicles do not form a coherent group either – for example, *motorcycle* which was paired with *bike* in both previous sets, now pairs with *train*, while *bike* goes with *stool*. Moreover, Figure 2C reveals that there is very little and very weak correlational structure in this data set.

We believe that this messy and weak conceptual organization can be explained by the data set’s low representational density and, relatedly, the inconsistent listing of features for some concepts but not others (even when the features are relevant). This notion is supported by comparing the structure seen in this original data set with the edited version (Figures 1D and 2D). Figure 2D shows a clear correlational structure, especially within the animals but also within the fruits and vegetables, and more weakly within the artifacts (similar to the Rogers et al. and the Garrard et al. sets). Figure 1D exhibits a highly categorical organization, with the birds clustering together, and grouping with the rest of the animals, and the fruits and vegetables clustering together and attaching to the artifacts. The vehicles also cluster together (with the exception of *sled*) as do the containers (*barrel*, *basket*, and *dustbin*). Similarly to the Rogers et al. set, the animal group exhibits taxonomic substructure with four distinct categories – large land animals, small land animals, canine/felines, and amphibian/reptiles.

Overall, the perceptual representations show a relatively consistent and generally categorically organized conceptual structure – which is driven not by knowledge of categories (given that category is not coded directly in the feature vectors) but by the sheer fact that members of specific taxonomic groups tend to share perceptual attributes.

#### Functional representations

**Figure 3:**
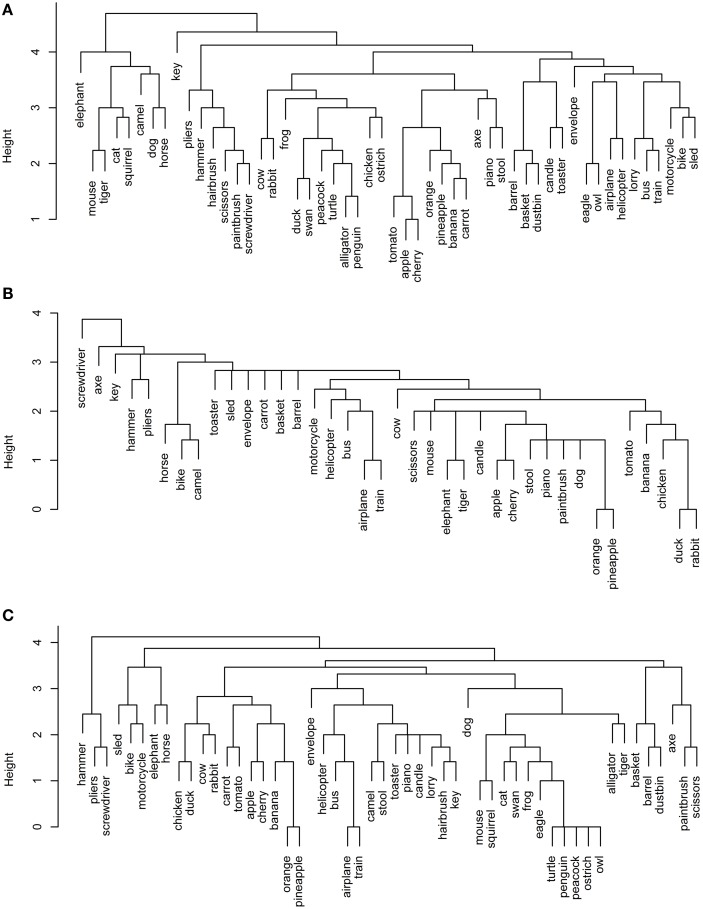
**Dendrograms from hierarchical cluster analysis for each functional data set: (A) Garrard et al. functional; (B) Cree and McRae functional; (C) Cree and McRae edited functional**.

**Figure 4:**
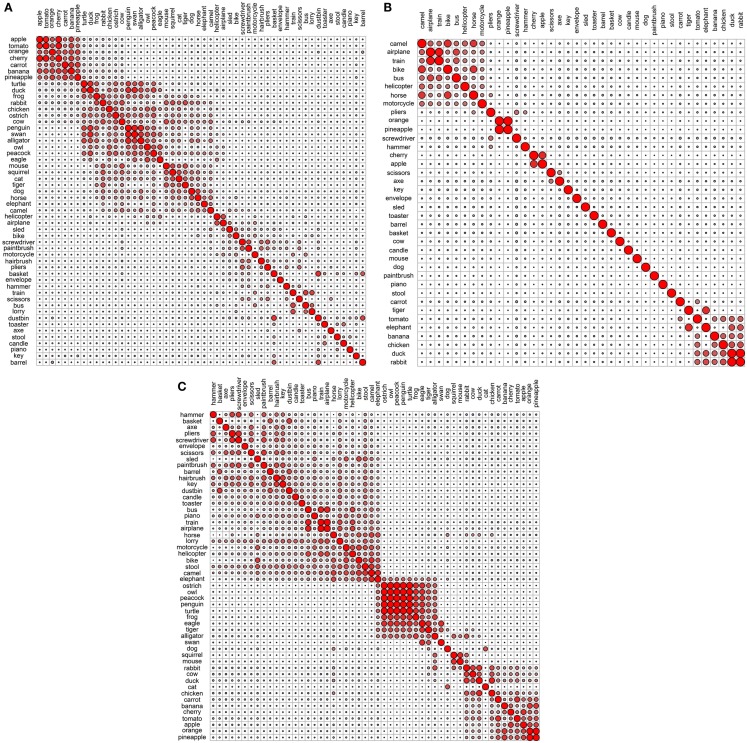
**Correlational plots for each functional data set: (A) Garrard et al. functional; (B) Cree and McRae functional; (C) Cree and McRae edited functional**.

The first thing to note about the functional representations (Figures 3 and 4) is that their organization is much flatter than that of the perceptual ones. While the members of some taxonomic categories do cluster together (like fruits and vegetables, vehicles, containers, tools), members of other categories are often present in these clusters too, and there is considerably less of the categorical substructure that was observed with the perceptual representations (e.g., the birds as a subcategory of animals).

In the Garrard et al. functional set, the fruits and vegetables cluster together and are strongly intercorrelated (Figures 3A and 4A). Most of the tools form a cluster as well, as do the three containers. Eight of the animals form a separate group while the other five join the birds group. Interestingly, the two water-inhabiting reptiles (*alligator* and *turtle*) pair with *penguin* (a water-inhabiting bird) and then attach to the other birds. Another curious observation is that *eagle* and *owl* (the two most prominent flyers among the birds) pair with *airplane* and *helicopter*. The other six vehicles form their own cluster as well.

The Cree and McRae functional set has the flattest dendrogram (Figure 3B) and a very weak correlational structure (Figure 4B). There are two notable groups – food, including *rabbit*, *duck*, *chicken*, *banana*, and *tomato*, which intercorrelate and also form a single cluster; transportation, including six of the eight vehicles and a pair animals (*camel* and *horse*). In the dendrogram, these items formed two separate clusters (*horse*, *bike*, and *camel* vs. *motorcycle*, *helicopter*, *bus*, *airplane*, and *train*). In addition, *orange* and *pineapple* are strongly correlated, as are *cherry* and *apple*.

The edited version of the Cree and McRae set has a much more pronounced correlational structure (Figure 4C). The food group has expanded to include all the fruits and vegetables as well as *rabbit*, *duck*, *chicken*, and *cow*. The artifacts all correlate together, with the transportation subcategory now including all eight vehicles and three animals (*camel*, *horse*, and *elephant*), as well as *stool* – which even though is not a means of transportation is functionally related because it is something we sit on. A group of relatively rare animals and birds also correlate together strongly.

The dendrogram shows a much richer structure than the original set as well (Figure 3C). As suggested by the correlational plot, the food items form a single coherent cluster as do the set of rare animals and birds (*dog* and *cat* group with these animals as well, even though they did not correlate with them). The intercorrelated transportation items formed two separate clusters (*bike*, *motorcycle*, *sled*, *horse*, and *elephant* vs. *train*, *airplane*, *bus*, and *helicopter*). In addition, the containers cluster together too, and the tools form two distinct clusters (*screwdriver*, *pliers*, and *hammer* vs. *axe*, *scissors*, and *paintbrush*).

Overall, even though we can talk about taxonomic categories within the functional representations, the concepts in these sets are clearly organized according to different principles compared to the perceptual representations – the organization here is driven by item behavior and use. For example, animals mostly group together because they do similar things (move, eat, etc.) and we do similar things with them (look at them, feed them, cook them, etc.). However, the few animals that have other uses (like food or transportation) cluster with different items, not with the animal group.

#### Encyclopedic representations

The encyclopedic features give rise to a conceptual structure again flatter than the one created by perceptual features, but it is also notably different from the functional organization. There tend to be smaller groups of pairs and triplets of related items. The clusters we see here generally obey the animal vs. non-animal distinction, and the fruits and vegetables tend to group together, as do the vehicles; but other than that, the organization is non-taxonomic. For example, as in the functional sets, birds are mixed with the other animals: not randomly but in interesting and predictable ways.

**Figure 5:**
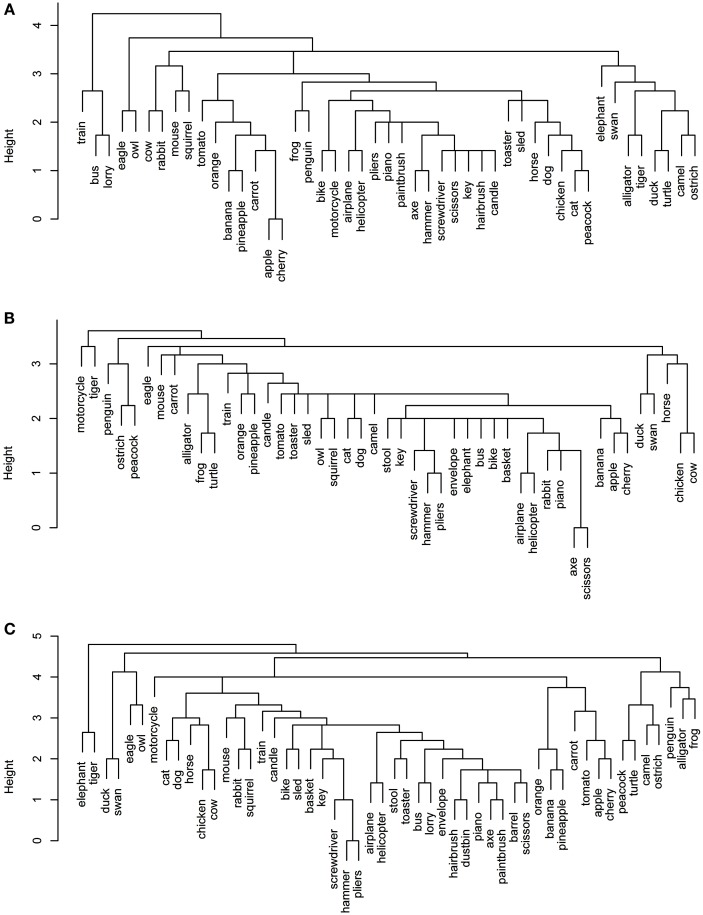
**Dendrograms from hierarchical cluster analysis for each encyclopedic data set: (A) Garrard et al. encyclopedic; (B) Cree and McRae encyclopedic; (C) Cree and McRae edited encyclopedic**.

**Figure 6:**
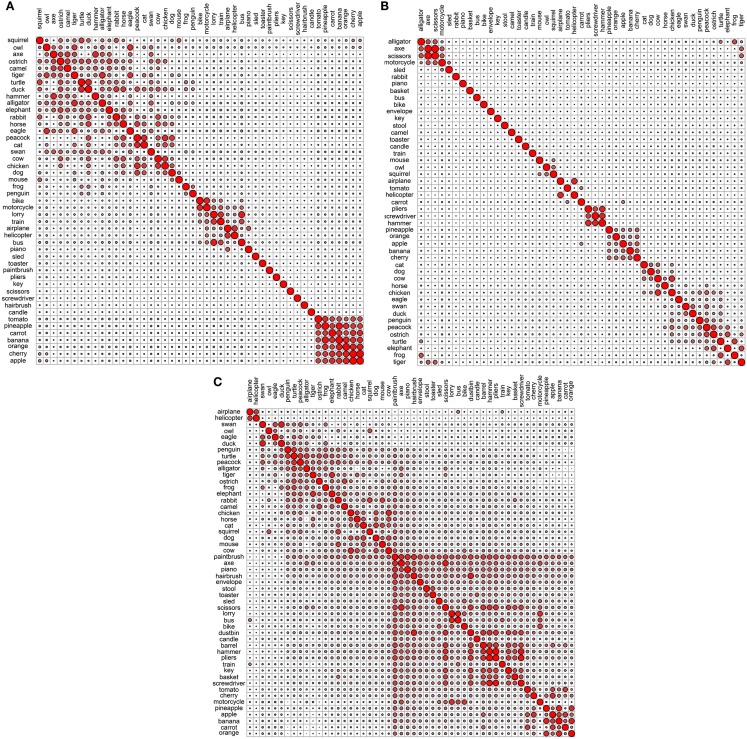
**Correlational plots for each encyclopedic data set: (A) Garrard et al. encyclopedic; (B) Cree and McRae encyclopedic; (C) Cree and McRae edited encyclopedic**.

In the Garrard et al. encyclopedic set (Figures 5A and 6A), the strongest grouping is that of the fruits and vegetables – they form a single cluster and intercorrelate strongly. In addition, the vehicles intercorrelate, but in the dendrogram they do not all group together – there is a cluster of the three large land vehicles (*train*, *bus*, *lorry*); a pairing of the smaller land vehicles (*bike* and *motorcycle*), and a pairing of the aircrafts (*airplane* and *helicopter*). The dendrogram shows a cluster of 10 household items including some tools, but the correlational plot demonstrates that, other than *axe* and *hammer*, which pair together, these items do not correlate with each other or with any other item; they are simply more or less idiosyncratic. Finally, the birds and animals mostly fall into two groups – domesticated farm animals (*horse*, *dog*, *chicken*, *cat*, and strangely *peacock*, which is also listed as domesticated), and a miscellaneous group of mostly rare animals and birds (*elephant*, *swan*, *alligator*, *tiger*, *turtle*, *camel*, *ostrich*, and oddly *duck*) – these are all species one may see at the zoo.

As with the original Cree and McRae functional set, the encyclopedic set exhibits a relatively flat dendrogram and weak correlational structure (Figures 5B and 6B). The fruits and vegetables are intercorrelated but they do not group together in the dendrogram. There are a few smaller clusters including a triplet of tools (*pliers*, *screwdriver*, and *hammer*), a group of reptiles/amphibians, a group of non-flying birds (*ostrich*, *peacock*, and *penguin*) and a group of five farm animals and birds. Even though the birds appear in different areas of the dendrogram, they are all intercorrelated (and correlated to *turtle* as well). There also seem to be some random pairings (e.g., *motorcycle* and *tiger*), though those do not seem to be supported by the correlational plot.

In the edited Cree and McRae encyclopedic set (Figures 5C and 6C), all the fruits and vegetables are not only intercorrelated but also cluster together. There is also a more coherent group of domesticated farm animals and birds (*cat*, *dog*, *horse*, *cow*, and *chicken*). The triplet of tools that we observed in the original set is still present. The flying birds (*duck*, *swan*, *eagle*, and *owl*) cluster together, while the non-flying birds group with an interesting set of animals (for example, *penguin* goes with two other water inhabitants, *alligator* and *frog*, while *ostrich* goes with *camel*). As in the original set, the birds correlate with each other, with *turtle*, and with *alligator*. It may seem strange that birds group with reptiles/amphibians but in addition to shared habitats, these two classes also share laying eggs.

Overall, the encyclopedic representations give rise to a relatively flat and more localized structure with smaller groups of items organized by relational principles such as where you normally see these items (at home, in a toolbox, on a farm, in the garden, on the road, in the sky, in the water, in the desert, and so on).

#### Verbal representations

**Figure 7:**
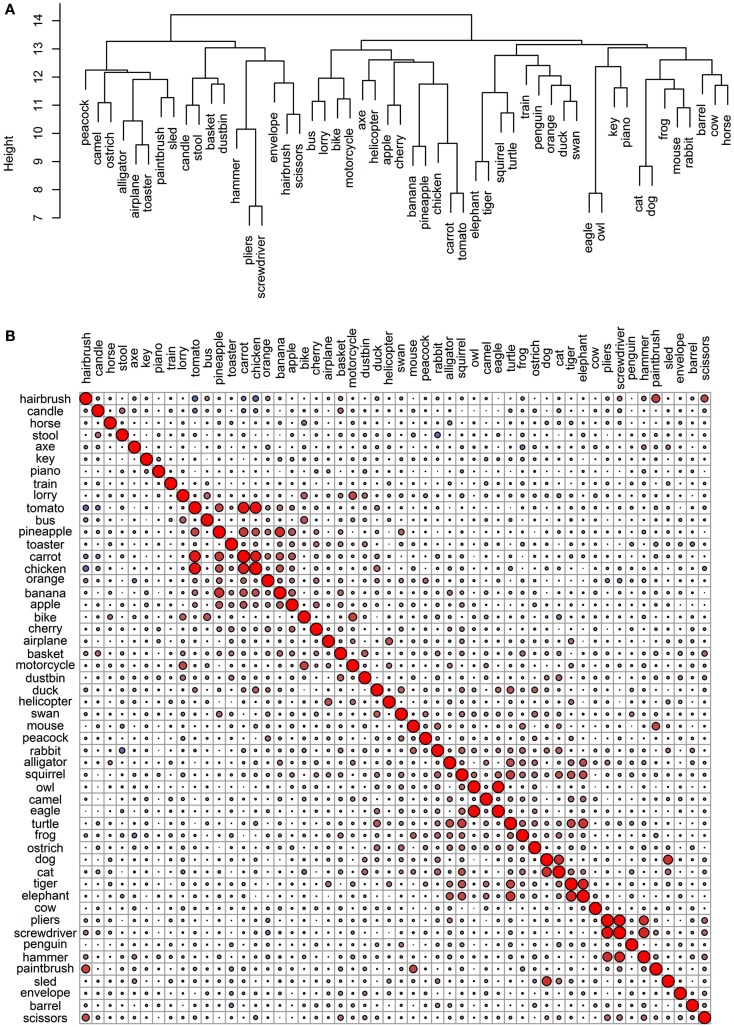
**Conceptual organization of the Hoffman et al. verbal data set: (A) hierarchical cluster analysis dendrogram; (B) correlational plot**.

The 52 verbal representations derived by Hoffman et al.’s LSA are very weakly intercorrelated (Figure 7B) and seem to be the set least organized by taxonomy (Figure 7A). There is no general differentiation between animal and non-animal items (as we saw in the previous sets). Nonetheless, there are some smaller categorical clusters. For example, a pair of birds (*eagle* and *owl*) and a triplet of tools (*pliers*, *screwdriver*, and *hammer*) group together and strongly intercorrelate. The four road vehicles (*bus*, *lorry*, *bike*, and *motorcycle*) also go together. *Airplane* and *helicopter* correlate, but they do not pair together in the dendrogram; *train* does not correlate with any of the other items, and interestingly, *sled* correlates most strongly with *dog* (though they do not pair together, because *dog* correlates yet more strongly with *cat*). Finally, there is a coherent cluster of food items including six of the fruits and vegetables as well as *chicken*. These items intercorrelate as well.

The dendrogram also includes a group of animals one may see on a farm (*horse*, *cow*, *dog*, *cat*, *mouse*, *rabbit*, and oddly *frog*), with the addition of *barrel* (another item not uncommon in this context). This cluster, however, is not supported by the correlational plot (other than the pairing of *dog* and *cat*, which correlate strongly). In addition to *dog* and *sled*, there are a few other interesting pairings in this set, including *elephant* and *tiger*, *hairbrush* and *scissors*, and *piano* and *key*. While one might argue that the similarity between elephant and tiger and perhaps even hairbrush and scissors is categorical in nature (perhaps due to perceptual, encyclopedic, or functional commonalities), it is crystal clear that the similarity between dog and sled and piano and key is of a different – associative – nature.

Overall, the hierarchical cluster analysis and the pairwise correlations of the verbal representations illustrate that the principle governing the conceptual organization here is contextual similarity, as would be expected given the origin of these representations. Sometimes, this may be consistent with functional attributes (as items that do similar things and are used in similar ways or for similar purposes tend to appear in common verbal contexts); other times, it may be consistent with encyclopedic attributes (e.g., location, origin, behavior); and in yet other cases, it may have an associative or relational nature (e.g., *dog* and *sled*; *piano* and *key*; *hairbrush* and *scissors*).

As noted at the beginning of the section, in a final effort to assess the representational structure present in each modality-specific feature type, we investigated the most similar items for all concepts. To do this, we used cosine as a standard measure of similarity between two *n*-dimensional vectors, *a* and *b*: $cos=\frac{\sum_{i=1}^{n}\left(a_{i}b_{i}\right)}{\sqrt{\sum_{i=1}^{n}\left(a_{i}^{2}\right)\sum_{i=1}^{n}\left(b_{i}^{2}\right)}}.$ The higher the cosine, the higher the similarity. This analysis progressed as follows: (1) calculate the cosine of each pair of vectors in a given set; (2) for each concept, calculate the average cosine value and its standard deviation; (3) for each concept, generate a list of items whose cosine with the target concept is at least two standard deviations above the average for that target concept. In other words, the resulting lists indicated items that were exceptionally similar to the target concept. The full set of lists is included in Appendix. Table 3 shows 10 concepts (two from five categories) chosen to illustrate the salient and important differences of the conceptual structure across modalities.

**Table 3:** **Lists of items most similar to a set of 10 concepts in each of the 11 data sets**.

**(A)**						
Concept	Rogers et al. visual	Garrard et al. sensory	Cree and McRae perceptual		Cree and McRae edited perceptual	Hoffman et al. verbal
Chicken	Peacock, owl, eagle, ostrich	Duck, eagle, owl	Eagle, duck, owl, swan		Peacock	Tomato, carrot

Duck	Swan, chicken, peacock, ostrich	Chicken, penguin	Swan, penguin, eagle, chicken, owl		Swan	Turtle, swan, chicken, ostrich

Cow	Camel, horse, dog	Horse, camel	Horse, elephant		Horse, camel	Elephant

Horse	Cow, camel	Mouse, dog	Elephant, squirrel, cat		Cow, camel	Bike

Carrot	Banana, pineapple, candle, cherry	Apple, cherry, tomato, orange, banana	Paintbrush, screwdriver, banana, alligator, orange, tiger		Orange, banana, cherry, apple, pineapple	Tomato, chicken, pineapple

Apple	Tomato, orange, banana, pineapple	Cherry, tomato, carrot, orange, banana, pineapple	Tomato, cherry, pineapple, orange		Tomato, cherry, pineapple, banana	Pineapple, carrot, banana

Bus	Lorry, train, airplane	Helicopter, airplane, train, motorcycle	Motorcycle, lorry, train		Train, airplane, helicopter, lorry, motorcycle	Bike, lorry

Bike	Motorcycle, screwdriver	Motorcycle, sled, bus	Motorcycle, train, sled		Motorcycle, sled	Motorcycle, bus, lorry

Screwdriver	Paintbrush, hammer, axe	Axe, hairbrush, scissors	Hairbrush, hammer, paintbrush, axe		Hammer, paintbrush, axe	Pliers, hammer

Pliers	Scissors, orange, apple	Key, hammer, axe	Scissors		Hairbrush, sled	Screwdriver, hammer

**(B)**						
**Concept**	**Garrard et al. functional**	**Cree and McRae functional**	**Cree and McRae edited functional**	**Garrard et al. encyclopedic**	**Cree and McRae encyclopedic**	**Cree and McRae edited encyclopedic**

Chicken	Owl, peacock, ostrich	Duck, rabbit, banana, tomato	Duck, cow, rabbit	Peacock, cow, cat	Cow, peacock	Cow, dog, horse

Duck	Swan, penguin, turtle, peacock	Chicken, elephant, banana, tomato	Chicken, rabbit	Turtle, rabbit	Swan, turtle, peacock	Swan, frog, turtle

Cow	Mouse, rabbit	NONE	Rabbit, chicken, duck	Chicken, rabbit, horse	Chicken, cat, dog	Chicken, dog, horse

Horse	Dog, camel	Bike, camel	Camel, elephant, bike	Duck, peacock, cow	Cow, peacock, chicken, swan	Cow, chicken, cat, dog

Carrot	Banana, pineapple, orange	Tomato	Tomato, orange, pineapple	Apple, banana, cherry, pineapple, orange, tomato	Apple, tomato	Apple, tomato

Apple	Cherry, tomato, banana	Cherry	Cherry, orange, pineapple, tomato	Orange, banana, carrot, pineapple, tomato	Cherry, banana, orange, pineapple, carrot	Cherry, carrot, banana, tomato

Bus	Train, lorry, motorcycle	Airplane, train	Airplane, train, helicopter	Lorry, train	NONE	Lorry, motorcycle, paintbrush

Bike	Sled, motorcycle	Camel, horse	Camel, motorcycle, sled, horse	Motorcycle, lorry, train	NONE	Motorcycle, lorry, paintbrush, sled

Screwdriver	Paintbrush, pliers, hairbrush	Pliers, hammer	Pliers, hammer, paintbrush, scissors	NONE	Hammer, pliers	Hammer, pliers

Pliers	Screwdriver, scissors, paintbrush, hammer	Screwdriver, hammer, horse	Hammer, screwdriver, paintbrush, scissors	NONE	Hammer, screwdriver	Screwdriver, barrel, dustbin, scissors

*(a) Similarity lists for the sensory/perceptual and the verbal data sets. (b) Similarity lists for the functional and encyclopedic data sets*.

*Chicken* is perceptually most similar to other birds including *peacock*, *eagle*, and *owl*. It is functionally most similar to other birds that don’t fly like *peacock* and *ostrich* (in the Garrard et al. set) as well as other animals we cook and eat like *duck* and *rabbit* (in the Cree et al. set). Encyclopedically, *chicken* is most similar to other animals domesticated farm animals (*cow*, *cat*, *dog*, *horse*) and *peacock* (which was also listed as domesticated), while verbally it is most similar to other things we eat and/or cook together with chicken, namely vegetables (*carrot* and *tomato* are the only two vegetables on our concept list). Likewise, *duck* is perceptually most similar to *swan* and *chicken*; functionally most similar to other animals that swim like *swan*, *penguin* and *turtle* (in the Garrard et al. set) as well as other animals we eat like *chicken* and *rabbit* (in the Cree et al. set). Encyclopedically, *duck* is most similar to other animals that like water (*turtle*, *swan*, *frog*); and verbally, it is also most similar to animals in the water (*turtle* and *swan*) but also other birds (*chicken* and *ostrich*).

These two examples draw attention to a discrepancy in the feature labeling between the Garrard et al. set and the Cree and McRae set. While the former included behavioral characteristics (like flying, swimming, laying eggs) in the functional knowledge type, the latter set classified those as encyclopedic features. Hence the overlap in similarity we see here.

The next pair of items are two farm animals, *cow* and *horse*. Perceptually, they are similar to each other as well as to *camel* (which is another hooved animal of similar size). Functionally, *horse* is most similar to other items that can be used to ride on like *camel*, *bike*, and *elephant*, as well as *dog* (both horses and dogs are used to pull things). *Cow*, on the other hand, is most similar to other animals we cook and eat like *rabbit*, *chicken*, and *duck* (in the Cree and McRae set); in the Garrard et al. set, it appears as most similar to *mouse* and *rabbit*, but this again is an artifact of the varying classification [in addition to being edible – which is something *cow* shares with *rabbit* – the three animals share the ability to breed, chew, eat, walk, and run, which are all features that would be classified as perceptual or encyclopedic in nature according to Cree and McRae (2003)]. Encyclopedically, cow and horse are similar to each other as well as other domesticated farm animals (*chicken*, *dog*, and *cat*). Verbally, the two differ as well – *horse* tends to appear in verbal contexts similar to *bike* (probably due to their shared function), while *cow* tends to appear in verbal contexts shared with *elephant* (this latter finding was somewhat surprising).

The next pair of examples comes from the fruit-and-vegetable category. Perceptually, *carrot* is most similar to same category members with common shape and/or color (*banana* and *orange*) as well as other items with an elongated shape. Likewise, *apple* is most similar to *tomato*, *orange*, *cherry*, and *pineapple*. Functionally, they also relate most highly to same category members, notably *carrot* is most similar to the other vegetable in the set (*tomato*) as well as some fruit we use to make juice (*orange* and *pineapple*), while *apple* is most similar to *cherry* (both being fruits we grow on trees in our gardens and pick, and use to make pie!) Encyclopedically, both *carrot* and *apple* have most in common with other things we commonly grow in our gardens (each other, as well as *tomato*); in addition, *apple* is similar to other fruits that grow on trees (*cherry*, *banana*, *orange*, and *pineapple*). Verbally, both items share contexts with their own sets of food items (*tomato*, *chicken*, and *pineapple* for *carrot*; *pineapple*, *carrot*, and *banana* for *apple*).

Moving to the artifacts, *bus* and *bike* are most similar to other vehicles in all four feature types but in subtly different ways. Perceptually, *bus* shares the most with other large vehicles (*lorry*, *train*, and *airplane*), while *bike* is most similar to the smaller vehicles (*motorcycle* and *sled*). Functionally, *bus* is most similar to other vehicles used for public transportation (*train* and *airplane*), while *bike* is most similar to other things we can sit on and ride – not only vehicles (*motorcycle* and *sled*) but also animals (*camel* and *horse*). Encyclopedically, both *bus* and *bike* have most in common with other vehicles seen on the road (*lorry* and *motorcycle*), which also seems to be the most commonly shared verbal context (as *bus* is verbally most similar to *bike* and *lorry*, and *bike* is verbally most similar to *motorcycle*, *bus*, and *lorry*). *Bike* and *motorcycle* have a very high verbal similarity also due to the fact that they are often used synonymously.

The final example is a pair of tools, which – like the vehicles – are most similar to other items from their category in all four knowledge types. Perceptually, *screwdriver* has most in common with *hairbrush*, *hammer*, and *axe* (they all have a single handle and, with the exception of axe, similar size); likewise, *pliers* has most in common with *scissors*. Functionally, both items are very similar to implements used in a handheld manner for handiwork (*screwdriver*, *pliers*, *hammer*, and *paintbrush*); in addition, *pliers* are like *scissors* in that they can cut. Encyclopedically as well as verbally, *screwdriver* and *pliers* are also most similar to each other and *hammer* (which is another item commonly found in a toolbox), but not any of the other implements that appeared in the perceptual and functional sets.

In summary, this analysis supports the results obtained in the hierarchical cluster analyses and the correlations – the conceptual representations within the investigated four knowledge types organize in unique and sensible ways – within the perceptual modality, conceptual structure is governed by perceptual similarity (most prominently shape, size, color, and parts); within the functional modality, the structure is directed by similarity in use and interaction; within the encyclopedic modality, it obeys commonality in location, habitat, and/or behavior; within the verbal modality, it is associative or relational (similarity within the verbal domain may be functional or encyclopedic in nature but need not be).

### Comparing conceptual structure between modalities

The second step in our investigation was to determine how conceptual structure compares across modalities. To do this, we took the distance matrices for each set – that is, the set of Euclidean distances between each pair of concepts within a given data set (these same matrices were used in the hierarchical cluster analyses discussed above) – and computed the pairwise matrix correlations for all data sets. The analysis was done twice – first with the original Cree and McRae feature list and then with the edited version. The results are presented in Table 4.

**Table 4:** **Pairwise correlations between the distance matrices within each data set as well as compared to simple taxonomic structure and random structure**.

**(A)**									
	Garrard et al. sensory	Cree and McRae perceptual	Garrard et al. functional	Cree and McRae functional (*n* = 38)	Garrard et al. encyclopedic	Cree and McRae encyclopedic (*n* = 47)	Hoffman et al. verbal	Taxonomic structure	Random structure
Rogers et al. visual	0.55***	0.35*	0.27	−0.13	0.40**	0.26	0.06	0.40**	−0.03
Garrard et al. Sensory		0.52***	0.37*	−0.15	0.29	0.15	−0.05	0.60***	0.02
Cree and McRae perceptual			0.19	−0.32*	0.24	0.19	0.03	0.32*	0.01
Garrard et al. functional				0.11	0.14	0.01	0.30*	0.31*	0.01
Cree and McRae functional (*n* = 38)					−0.13	−0.16	−0.03	−0.04	−0.01
Garrard et al. encyclopedic						0.27	0.12	0.23	−0.06
Cree and McRae encyclopedic (*n* = 47)							0.07	0.1	−0.02
Hoffman et al. verbal								−0.03	−0.04

**(B)**									
	**Garrard et al. sensory**	**Cree and McRae edited perceptual**	**Garrard et al. functional**	**Cree and McRae edited functional**	**Garrard et al. encyclopedic**	**Cree and McRae edited encyclopedic**	**Hoffman et al. verbal**	**Taxonomic structure**	**Random structure**

Rogers et al. visual	0.55***	0.72***	0.27	0.08	0.40**	0.43**	0.06	0.40**	−0.03
Garrard et al. Sensory		0.65***	0.37*	0.10	0.29	0.42**	−0.05	0.60***	0.02
Cree and McRae edited perceptual			0.42**	0.13	0.36*	0.57***	0.22	0.46**	−0.01
Garrard et al. functional				0.33*	0.14	0.14	0.30*	0.31*	0.01
Cree and McRae edited functional					0.02	−0.03	0.13	0.28*	0.01
Garrard et al. encyclopedic						0.43**	0.12	0.23	−0.06
Cree and McRae edited encyclopedic							0.10	0.30*	−0.01
Hoffman et al. verbal								−0.03	−0.04

(a) With the original Cree and McRae feature lists; (b) with the edited Cree and McRae feature lists. ***p < 0.0005, **p < 0.005, *p < 0.05.

The most obvious finding is that the three sensory/perceptual sets intercorrelate. The correlations between the Cree and McRae set and the other two are greatly improved in the edited version, especially with the Rogers et al. set where the correlation doubles. There is also a notable trend for the conceptual structure of this type of representations to correlate with that of the encyclopedic knowledge type (Figure 4B), but much less so with the functional knowledge type. In fact, the original Cree and McRae perceptual and functional sets correlated negatively. The Garrard et al. functional set appears to organize more consistently with the one in the sensory modality, which probably has to do with the specific classification of features, whereby statements that had to do with behavior (e.g., lays eggs, swims, dives, runs, flies, jumps) were included in the functional feature list as opposed to the perceptual (motion) or encyclopedic feature lists, as was done by Cree and McRae. Finally, the conceptual structure present in the sensory/perceptual modality did not correlate at all with that in the verbal modality.

The conceptual networks in the functional and encyclopedic modality are considerably more idiosyncratic than that in the perceptual modality, as evident by the fewer significant correlations involving these sets (Table 4). Using the edited Cree and McRae sets, we found that the concepts in the two functional sets organize in similar ways (in fact, this was the only significant correlation involving the Cree and McRae functional structure). The same is true for the two encyclopedic sets. These two types of knowledge did not correlate with each other, though the functional modality had a tendency to relate to the structure in the verbal modality. Finally, as noted above, the structure within the encyclopedic modality also correlated with that in the perceptual modality.

The conceptual organization within the verbal modality appears to be most unique. The only significant correlation (and still pretty small in magnitude) was with the functional sets (Garrard et al. and the edited Cree and McRae). This finding is consistent with the observations we made based on the dendrograms, correlational plots, and item similarity lists.

In summary, by statistically comparing how concepts relate to each other in each data set, we established that the various sets, which came from different sources, are mostly consistent with each other (with the slight exception of the Garrard et al. functional list). Furthermore, we found how conceptual structure relates across modalities. The organization within the perceptual modality is somewhat similar to that in the encyclopedic modality, and both of those are different from the functional and verbal modality. There is also some similarity between the functional and verbal knowledge types, but generally, those appear to be organized in idiosyncratic ways.

### Comparing conceptual structure to simple categorical structure

**Figure 8:**
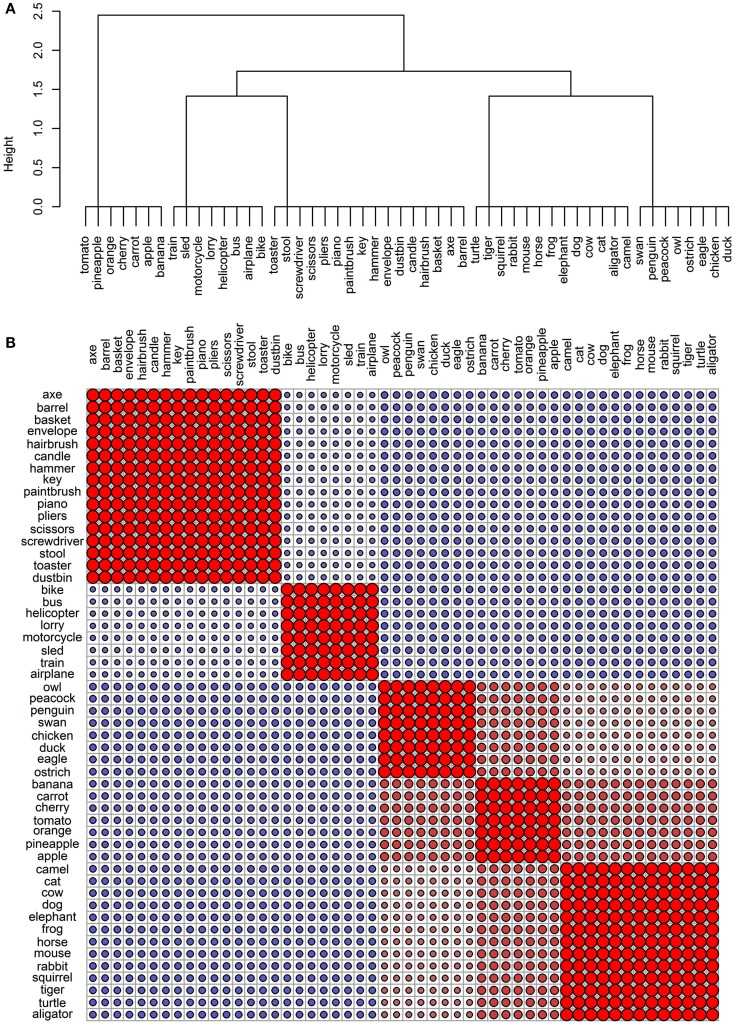
**Conceptual organization of a simple taxonomic structure: (A) hierarchical cluster analysis dendrogram; (B) correlational plot**.

The final goal of this investigation was to determine the degree to which the representational networks within each modality were taxonomically organized (i.e., organized according to category). To do this, we generated a simple “reference” categorical structure (Figure 8) where the animals, the artifacts, and the fruits and vegetables all form distinct clusters; the birds are a subcategory of the animals, and the vehicles are a subcategory of the artifacts. We computed the pairwise correlations of the distance matrix in this simple categorical structure and the distance matrices in the 11 data sets. As can be seen in Table 4, the sensory/perceptual modality is the only one that consistently correlated with the taxonomic structure, with the Garrard et al. sensory set having the highest correlation.

We then assessed whether the correlations between the sets and the taxonomic reference structure were significantly different from each other, taking into account the between-set correlations. The results are shown on Table 5 and they confirmed that taxonomic organization is most prominent in the Garrard et al. sensory set. The other correlations did not systematically differ from each other, with the exception of the verbal modality, where the lack of categorical organization was significantly different from all sets which had notable categorical structure (the four sensory/perceptual sets, the Garrard et al. functional set, and the Cree and McRae edited functional and encyclopedic sets).

**Table 5:** **Correlations between the distance matrices in each data set and the correlation matrix in a simple taxonomic structure**.

**(A)**								
Data set	*r* Value	Garrard et al. sensory	Cree and McRae perceptual	Garrard et al. functional	Cree and McRae functional (*n* = 38)	Garrard et al. encyclopedic	Cree and McRae encyclopedic (*n* = 47)	Hoffman et al. verbal
		0.60***	0.32*	0.31*	−0.04	0.23	0.1	−0.03
Rogers et al. visual	0.40**	√			√		√	√
Garrard et al. sensory	0.60***		√	√	√	√	√	√
Cree and McRae perceptual	0.32*							√
Garrard et al. functional	0.31*							√
Cree and McRae functional (*n* = 38)	−0.04							
Garrard et al. encyclopedic	0.23							
Cree and McRae encyclopedic (*n* = 47)	0.1							

**(B**)								
**Data set**	***r* Value**	**Garrard et al. sensory**	**Cree and McRae edited perceptual**	**Garrard et al. functional**	**Cree and McRae edited functional**	**Garrard et al. encyclopedic**	**Cree and McRae edited encyclopedic**	**Hoffman et al. verbal**
		0.60***	0.46**	0.31*	0.28*	**0.23**	0.30*	**−0.03**

Rogers et al. visual	0.40**	√						√
Garrard et al. sensory	0.60***			√	√	√	√	√
Cree and McRae edited perceptual	0.46**							√
Garrard et al. functional	0.31*							√
Cree and McRae edited functional	0.28*							√
Garrard et al. encyclopedic	0.23							
Cree and McRae edited encyclopedic	0.30*							√

*Ticks mark values that are significantly different from each other (*p* < 0.05), (a) With the original Cree and McRae feature lists; (b) with the edited Cree and McRae feature lists*.

### Comparing conceptual structure to random structure

In the modalities where concepts are not organized taxonomically, is the structure random? To assess this, we compared the distance matrices to 1000 random permutations of the distance matrix from the taxonomic structure. The average correlation values are presented in the last column of Figure 4 and none of them are significant, confirming our previous observation that even though concepts do not group according to category in all modalities, there is coherent structure in each case.

## Discussion

This study employed a number of different data sets and analytical methods in order to assess the structure of information arising in four modalities of knowledge: perceptual, functional, encyclopedic, and verbal. We had three distinct goals in mind: (1) to establish the organization in each modality; (2) to compare the structure between the various modalities; and (3) to assess the degree to which each structure is taxonomic or random.

In summary, our results showed that there is abundant structure in each of the four modalities we investigated (none of it is random) but the organization differs across modalities. The visual/perceptual domain is the most hierarchically organized and closest to classic taxonomic structure. Items group into categories and subcategories based on their prominent sensory characteristics (most importantly, shape, size, color, and parts). The organization in this modality is measurably different from the one in the functional modality, where concepts organize according to experience of interaction and use. Generally, this does *not* correlate with perceptual experience, though *occasionally* it might (e.g., in the case of some tools, which are visually as well as functionally similar). Encyclopedic knowledge gives rise to yet another conceptual organization, governed by experience of shared location or behavior. This type of structure appears to correlate with the organization within the perceptual domain (at least for this set of items) but not the functional domain. Finally, the verbal modality has the most unique structure, not at all categorical but also not random. It centers on associative or relational knowledge. It weakly resembles functional organization but notably deviates from perceptual and/or encyclopedic organization.

The findings from the current and previous studies all underline the fact that concepts are formed from a rich multimodal (verbal and non-verbal) set of experiences, where concepts relate to each other and organize in distinct ways. This, in turn, raises the question of how all these modality-specific experiences are fused together into coherent cross-modal conceptual knowledge which is capable of appropriate generalization across exemplars.

Some contemporary and classical theories postulate that the semantic system is simply this distributed network of modality-specific representations, all connected to one another (e.g., Eggert, 1977; Martin, 2007). Each modality-specific element within this distributed network would be able to code the local statistics (information structure) arising in that modality. There is a clear danger, however, that this system alone would lack knowledge of feature co-occurrence statistics across modalities (e.g., things that have beaks usually can fly, they lay eggs, nest in the trees, and sing songs). Without knowledge of the cross-modal coherent covariation of information, the semantic system would be unable to pull together the correct subset of cross-modal features for each concept and to generalize this information appropriately across concepts (Smith and Medin, 1981; Wittgenstein, 2001; Rogers and McClelland, 2004; Lambon Ralph et al., 2010). Extracting this kind of statistics is more than a simple linear summation of the individual modalities or learning pairwise correlations (see Rogers and McClelland, 2004, for a computational demonstration; and Lambon Ralph et al., 2010, for further discussion).

In keeping with these observations, a recent investigation employed a graph-theoretic approach to look at the relative contribution of perceptual and functional knowledge (based on Cree and McRae’s feature lists) to the conceptual organization of 130 common nouns, all acquired by 30 months of age (Hills et al., 2009). Hills and colleagues constructed three types of conceptual network – using the full set of features, using only the perceptual features, and using only the functional features. By calculating the average clustering coefficient for nodes in each network (that is, their tendency to share features with neighboring nodes), they evaluated the existence of structure in the networks (groups and subgroups of nodes). Their results closely resembled what we found here – there was significant structure (compared to random) in all three networks; the perceptual network was much denser than the functional network and clusters in the latter were smaller in size. Like ours, their results also indicated that these two types of features contribute differently to category organization. Furthermore, Hills et al. showed that despite the differences in the organization of these two modalities, the two types of features have a high degree of correspondence (or coherent covariation), creating conceptual structure above and beyond the structure existing in each one modality.

Recently, a number of investigations have focused on how verbal knowledge can be combined with feature type knowledge. For example, Steyvers (2010) presented a probabilistic model in which a text-based data-driven approach to extracting semantic information is augmented with knowledge of perceptual, functional, and encyclopedic features for a set of 287 animate and inanimate concepts. The results showed that the addition of feature information improved the model’s ability to generalize. Similarly, Durda et al. (2009) reported a neural network model trained with 445 concepts to map from textual co-occurrence vectors (similar to the verbal representation analyzed in our study) to feature representations based on the Cree and McRae norms. Like Steyver’s model, this model also exhibited a notable ability to generalize to novel concepts (i.e., ones it had not been trained on).

In an impressive computational modeling study, Andrews et al. (2009) adopted a probabilistic approach to extract semantics from a data set including both verbal and non-verbal (i.e., feature) information. The authors referred to these two types of information as distributional and experiential, respectively, to emphasize the point that language-based knowledge is qualitatively different from sensory-functional type of knowledge. Their distributional data set included about 8,000 short texts from the British National Corpus (each 150–250 words long), while their experiential data set included feature norms for 456 concepts. The model was trained using either set alone, the two sets in conjunction, or the two sets independently. In line with our findings, Andrews and colleagues observed that the semantic structure learned from the two types of information is markedly distinct, and further distinct from (and not as rich as) the structure arising when the two types of information are combined. Notably, they found that the structure in the two models trained with a single data set correlated higher with the structure in the model trained with the two sets independently than the one trained with the two sets jointly, suggesting that the conceptual organization arising under simultaneous exposure to multiple information sources is unique and different from the one arising from a single source or a linear combination of the multiple sources.

To assess performance, the model’s learned semantic similarity between concepts was compared to human behavioral data including lexical substitution errors, word-association norms, lexical priming, and semantic errors in picture naming. The results showed that the combined model was most similar to the behavioral data. The authors discussed the importance of both verbal and non-verbal information in the acquisition of semantic knowledge, and emphasized the point that cross-modal semantic representations rely on exposure to the statistical structure (what we earlier called coherent covariation) both within and *between* modalities.

All these findings and observations imply that additional computational machinery is required to fuse modality-specific information together to form coherent concepts. One possibility is provided by the hub-and-spoke account (e.g., Rogers et al., 2004; Patterson et al., 2007; Lambon Ralph et al., 2010; Pobric et al., 2010) which inherits the basic premise that the multiple verbal and non-verbal modalities provide the raw ingredients for the formation of concepts (they are the “spokes” of the semantic system) but there is an additional component (the “hub” of the system) that mediates between the various modalities. The representations learned in the hub are based on *complex non-linear mappings* among the modality-specific representations in the spokes (Wittgenstein, 2001; Lambon Ralph et al., 2010). Just as modality-specific knowledge pools have been localized to distinct areas in the brain, so has the proposed transmodal representational hub – with the anterior inferolateral temporal area being one crucial region. The clearest neuropsychological example of this comes from investigations of semantic dementia, where the patients’ maximal damage in this region leads to multimodal yet selective semantic impairment (Warrington, 1975; Snowden et al., 1989; Hodges et al., 1992; Bozeat et al., 2000). Convergent evidence for the importance of this region in semantic representation has been provided by functional imaging and repetitive transcranial magnetic stimulation studies with neurologically intact individuals (e.g., Vandenberghe et al., 1996; Marinkovic et al., 2003; Pobric et al., 2007; Binney et al., 2010; Visser and Lambon Ralph, 2011; Peelen and Caramazza, 2012; Visser et al., 2012). In addition, the location and connectivity of the transmodal hub has recently been established using diffusion-weighted tractography in neurologically intact participants (Binney et al., 2012) and its breakdown in semantic dementia (Acosta-Cabronero et al., 2011).

Two aspects of the hub-and-spoke theory are crucial. The first is that the transmodal hub provides the neural machinery to compute the non-linear mappings required for the formation of coherent concepts and their generalization on the basis of semantic rather than superficial (modality-specific) similarities. Indeed, recent targeted investigations of semantic dementia have shown that, in both verbal and non-verbal domains, the patients lose the coherence of these concepts and thus exhibit over- and under-generalization errors (Lambon Ralph and Patterson, 2008; Lambon Ralph et al., 2010; Mayberry et al., 2011). The second crucial aspect of this theory is that semantic representations require the combination of transmodal (hub) *and* modality-specific information sources. It is not, therefore, an issue of debating whether semantic representations are underpinned by hub OR spokes but rather how these work together to form coherent concepts. The importance of both elements is indicated in recent functional neuroimaging studies (e.g., Visser and Lambon Ralph, 2011; Peelen and Caramazza, 2012; Visser et al., 2012) and confirmed by probing hub-and-spoke regions in the same participants using rTMS (e.g., Pobric et al., 2010). In such circumstances, transient suppression of the transmodal ATL hub generates a pan-category semantic impairment whereas stimulation of the dorsal aspects of the inferior parietal lobule generates a category-specific impairment for manmade objects that relates directly to the suppression of praxis information that is coded in this region.

A recent behavioral paper focused around the issue of how concept-relevant information from different modalities is combined into coherent and useful cross-modal semantic representations (McNorgan et al., 2011). The authors distinguished between two types of theories: (1) what they called “shallow” theories, which postulate either direct connectivity between modality-specific representations (i.e., distributed multimodal semantics) or a connectivity of those areas into a single mediating construct (a cross-modal semantic hub); and (2) “deep” theories, which postulate a hierarchy of mediating constructs (convergence zones), which progressively combine modality-specific knowledge into increasingly more cross-modal representations (higher order modality-specific areas, bi-modal areas, tri-modal areas, and so on). Note that this classification would include both the multimodal semantic models and the hub-and-spoke framework discussed above under the “shallow” classification. The assumption is that the two types of models make different predictions about the processing time required to integrate information coming from a single modality vs. the processing time required to ingrate information coming from multiple modalities. They used four verbal feature verification tasks and the results supported a deep model of semantics. One weakness of this study is the use of verbal stimuli. The unspoken assumption that these stimuli will in fact activate modality-specific representations (such as visual or functional), and only then propagate activation forward to convergence zones or any associative areas is not discussed and may in fact have distinct implications for the different theories. This combined with the lack of imagining data to complement the behavioral findings hinders the ability to make claims about the processing involved in the tasks: what modality-specific and association areas are involved, what are the temporal dynamics, and so on. Nonetheless, the investigation provides further support to the notion that the semantic system involves more than simply distributed modality-specific areas, and presents an interesting and useful approach to distinguishing between models of semantics.

Returning to the original objective of our study, the natural next step in establishing the structure of the semantic system is to inquire about the conceptual organization of cross-modal representations. We found that modality-specific pools of knowledge exhibit meaningful and unique structure. How does this structure compare to a cross-modal combined representation of this knowledge? And how do the various modalities contribute? A number of previous investigations (e.g., Andrews et al., 2009; Durda et al., 2009; Steyvers, 2010) seem to attribute a prominent role to verbal information − contrasting it with feature type information independent of its modality, as opposed to treating it as yet another modality of experience as we have done in our approach (see also, Plaut, 2002; Rogers et al., 2004, for similar approaches within a connectionist paradigm).

Last but not least, we will consider a few methodological issues and contributions from our current work. Feature listings have often been criticized in the past as an unreliable method to probe people’s semantic knowledge (e.g., Murphy and Medin, 1985; Rogers et al., 2004) for at least three reasons: (1) participants know much *more* about each concept than what they list in any one study (therefore, the lists are incomplete); (2) the features that participants give is a potentially *random* sample of their full knowledge (therefore, the lists are inconsistent/variable); and (3) the knowledge is probed *verbally* for all types of features (therefore, the information provided is filtered by vocabulary demands which may impact some modalities of knowledge more than others because the attributes in that domain do not have verbal labels or are hard to express, e.g., elements of praxis or non-verbal auditory sounds).

We found that one way to improve the quality of the feature listings was to consider all features listed (independent of concept) and to re-score each concept against each feature. This guards against (quite common) cases where participants generate a certain feature (e.g., “has a long neck”) for one concept (e.g., *swan*) but not for another (e.g., *peacock*), even though it applies to both. Also, features that describe identical or similar aspects of the concept (e.g., “has a box-like shape” and “looks like a square”) should be grouped together to minimize redundancy and improve concept overlap. Although laborious (especially if undertaken for more than the 52 concepts considered in this study), combining these two techniques counteracts the incomplete and variable nature of feature listings. In this study, for example, it greatly improved the representational density of the Cree and McRae data set, which in turn resulted in an improved and more informative emergent structure.

The present investigation also provided an insight into the third concern listed above. We found that conceptual representations based on verbally reported features, taken to provide information about non-verbal modalities, are distinctly different from conceptual representations based on verbal experience (i.e., using the concept names in context). The established similarity and coherence between perceptual representations based on feature norms (re-scored) and perceptual representations based on participants’ drawings further supported the notion that verbal feature listings provide a close approximation of modality-specific non-verbal knowledge.

Of course, we have only considered a handful of knowledge types. The fact is that experience in some modalities (olfactory, gustatory, tactile, etc.) may not be as easily verbalized as visual or motor experience. Some researchers have solved this problem by asking their participants to give a rating of how relevant each knowledge type is to a specific item, instead of listing features of various types (e.g., Gainotti et al., 2009; Hoffman and Lambon Ralph, 2012). Gainotti et al. (2009) presented college students with the pictures and names of 28 living things and 21 artifacts, and asked them to rate the familiarity of each item and to indicate (on a scale from 0 to 7) how relevant each source of modality-specific knowledge was in defining each item. The knowledge types included visual, auditory, tactile, olfactory, gustatory, motor/functional, and encyclopedic. The raw scores were transformed into percentages indicating the relative contribution of each modality to each concept. Their results indicated that olfactory and gustatory experience was significantly relevant only to the plant subcategory (fruits, vegetables, and flowers), whereas tactile experience was most relevant to the same categories as functional experience, namely artifacts such as tools, clothing, and furniture. Even though the clever methodology allowed the researchers to collect data for all modalities (even those where features or attributes may not be easy to report), the analysis suffers from the same assumption as the other studies discussed earlier – namely that there is categorical organization within each modality, which as we have established here is not the standard structure across modalities.

## Conclusion

This study looked at three distinct methods of probing modality-specific knowledge (feature listings, drawings, and verbal co-occurrence statistics) to assess the conceptual structure in four modalities: perceptual, functional, encyclopedic, and verbal. Unlike previous studies, we did not assume that taxonomic categories exist in each knowledge type. Instead, we utilized a data-driven approach to reveal distinct and logical organization of concepts in each modality. Only the perceptual modality consistently exhibited significant categorical structure. Verbal representations had the most idiosyncratic organization, weakly related to the functional representations and very dissimilar from the perceptual and encyclopedic representations, which were somewhat similarly organized. Thus, the semantic system draws from these rich and multifaceted modality-specific pools of knowledge, each with a complex representational structure, to form coherent transmodal representations.

## Conflict of Interest Statement

The authors declare that the research was conducted in the absence of any commercial or financial relationships that could be construed as a potential conflict of interest.

## Footnotes

^1^The high dimensional semantic space resulting from Latent Semantic Analysis is a mathematical representation of a large set of terms (words, phrases) and it is unique to the corpus used. In other words, for the selected corpus and restrictions applied (frequency of occurrence, part of speech, etc.), LSA represents each term as a vector. This vector has no meaning, other than in relation to other vectors in the same semantic space (that is, other terms from the corpus). The relationship between two terms can be quantified with any distance or similarity measure applied to the pair of representational vectors, such as the Euclidean distance or the cosine of the angle between the vectors.

## Appendix

### Full list of concepts and most similar items in each data set

#### Procedure

In each data set, the cosine between each pair of representational vectors was computed. The items included in the following lists have a cosine with the target concept at least two standard deviations above the average cosine value for that target concept. NONE = no items exceeded the threshold. NA = this target concept was not present in the set.

**Table A1:** **Lists for the four sensory/perceptual sets and the verbal set**.

Concept	Rogers et al. visual	Garrard et al. sensory	Cree and McRae perceptual	Cree and McRae edited perceptual	Hoffman et al. verbal
Airplane	Bus, lorry, train, helicopter	Helicopter, train, bus	Eagle, sled	Bus, helicopter, train, motorcycle	Helicopter, tiger

Alligator	Turtle, dog	Tiger, camel	Banana, rabbit, frog, turtle	Turtle	Turtle, elephant

Apple	Tomato, orange, banana, pineapple	Cherry, tomato, carrot, orange, banana, pineapple	Tomato, cherry, pineapple, orange	Tomato, cherry, pineapple, banana	Pineapple, carrot, banana

Axe	Screwdriver, paintbrush, hammer	Hammer, screwdriver	Hammer, screwdriver	Hammer, scissors, screwdriver	Hammer, sled

Banana	Pineapple, orange, apple, carrot	Tomato, apple, cherry, carrot	Tomato, apple, alligator, orange	Orange, apple, tomato, carrot	Pineapple, carrot, chicken

Barrel	Dustbin, envelope, banana, candle	Dustbin, axe	Dustbin, camel	Basket, dustbin	Rabbit

Basket	Toaster	Dustbin, hairbrush, stool	Paintbrush, hammer, hairbrush	Barrel, envelope, paintbrush, dustbin	Dustbin

Bike	Motorcycle, screwdriver	Motorcycle, sled, bus	Motorcycle, train, sled	Motorcycle, sled	Motorcycle, bus, lorry

Hairbrush	Paintbrush, screwdriver	Paintbrush, hammer, screwdriver	Paintbrush, sled, scissors, dustbin, screwdriver	Paintbrush, sled	Paintbrush, scissors

Bus	Lorry, train, airplane	Helicopter, airplane, train, motorcycle	Motorcycle, lorry, train	Train, airplane, helicopter, lorry, motorcycle	Bike, lorry

Camel	Cow, horse, dog	Cow, horse	Barrel, cow, horse	Horse, cow	Elephant, tiger

Candle	Pineapple, stool, carrot	Hairbrush	Envelope, paintbrush, pliers, sled	Pliers, envelope, paintbrush	Basket, stool

Carrot	Banana, pineapple, candle, cherry	Apple, cherry, tomato, orange, banana	Paintbrush, screwdriver, banana, alligator, orange, tiger	Orange, banana, cherry, apple, pineapple	Tomato, chicken, pineapple

Cat	Dog, squirrel, tiger	Mouse, squirrel, rabbit, dog	Horse, squirrel, dog, mouse, elephant	Dog, tiger	Dog, squirrel

Cherry	Pineapple, carrot, tomato	Tomato, apple, orange, carrot	Apple, tomato, dustbin	Apple, tomato, orange, pineapple	Apple, orange, toaster

Chicken	Peacock, owl, eagle, ostrich	Duck, eagle, owl	Eagle, duck, owl, swan	Peacock	Tomato, carrot

Cow	Camel, horse, dog	Horse, camel	Horse, elephant	Horse, camel	Elephant

Dog	Tiger, cat, cow	Horse, mouse, cat, squirrel	Cat, squirrel, horse, elephant	Cat	Cat, sled

Duck	Swan, chicken, peacock, ostrich	Chicken, penguin	Swan, penguin, eagle, chicken, owl	Swan	Turtle, swan, chicken, ostrich

Dustbin	Barrel, envelope, piano, candle	Barrel, basket	Hairbrush, stool, key, sled	Barrel, basket	Basket, cat, lorry

Eagle	Peacock, chicken, ostrich, owl	Owl, chicken, swan, ostrich	Chicken, duck, owl, swan, peacock	Owl	Owl

Elephant	Cow, dog, camel	Turtle	Horse, squirrel	Horse	Tiger, turtle, squirrel, alligator

Envelope	Barrel, dustbin	Bus, train	Candle, paintbrush, pliers, sled	Basket, dustbin	Dustbin, scissors, basket

Frog	Rabbit, alligator, mouse	Alligator	Turtle, cat, cow	Turtle	Turtle, rabbit

Hammer	Screwdriver, key	Axe, hairbrush	Axe, basket, screwdriver	Axe, screwdriver, hairbrush	Screwdriver, pliers

Helicopter	Airplane, bus, lorry	Airplane, bus, train	Scissors, train, hammer	Airplane, bus, train	Airplane, motorcycle, tiger

Horse	Cow, camel	Mouse, dog	Elephant, squirrel, cat	Cow, camel	Bike

Key	Hammer, screwdriver	Pliers, hairbrush, hammer, screwdriver	Dustbin, stool	Dustbin, stool	Piano

Lorry	Bus, train	Motorcycle, airplane, train	Bus, motorcycle, train	Bus, train, airplane, motorcycle, helicopter	Motorcycle, bike, bus

Motorcycle	Bike	Bike, sled, lorry	Train, bus, bike, lorry	Bike, airplane, bus, train, lorry	Bike, lorry

Mouse	Rabbit	Rabbit, squirrel, cat, horse	Squirrel, cat, rabbit	Rabbit, squirrel	Paintbrush, rabbit, frog

Orange	Apple, banana, tomato	Cherry, tomato, apple, carrot	Tomato, apple, cherry	Banana, tomato, cherry, carrot, apple, pineapple	Pineapple, carrot

Ostrich	Swan, chicken, eagle, peacock	Eagle, peacock, swan	Eagle, swan, chicken	Peacock, eagle	Duck, swan, squirrel, alligator, turtle

Owl	Chicken, peacock, eagle, penguin	Eagle, chicken	Eagle, swan, chicken, duck	Eagle	Eagle

Paintbrush	Screwdriver, hairbrush, axe, hammer	Hairbrush, axe	Basket, hairbrush, screwdriver	Hairbrush, basket, sled	Hairbrush, mouse

Peacock	Chicken, eagle, owl, ostrich	Ostrich, chicken, duck, eagle, owl	Eagle, owl, swan	Ostrich, swan, duck	Swan, orange

Penguin	Owl, chicken	Duck, swan, chicken, eagle	Duck, swan	Duck, swan	Swan, cat

Piano	Dustbin, stool, sled	Barrel, hammer	Barrel, cow	Bus	Key, hammer

Pineapple	Tomato, banana, apple	Apple, cherry, tomato, carrot, banana, orange	Apple, orange	Apple, cherry, orange, tomato	Banana, carrot, tomato, chicken

Pliers	Scissors, orange, apple	Key, hammer, axe	Scissors	Hairbrush, sled	Screwdriver, hammer

Rabbit	Squirrel	Mouse, cat, squirrel	Mouse, alligator	Squirrel, mouse	Cat, squirrel, mouse

Scissors	Pliers, key, orange	Screwdriver, axe	Hairbrush, pliers, sled, helicopter, dustbin	Axe, hairbrush, pliers, sled	Hairbrush, pliers, screwdriver, hammer

Screwdriver	Paintbrush, hammer, axe	Axe, hairbrush, scissors	Hairbrush, hammer, paintbrush, axe	Hammer, paintbrush, axe	Pliers, hammer

Sled	Stool, piano, bus	Motorcycle, bike	Hairbrush, scissors	Hairbrush, paintbrush	Dog, cat

Squirrel	Rabbit, cat	Mouse, cat	Horse, mouse, elephant, cat	Rabbit, mouse, cat	Turtle, elephant, tiger

Stool	Sled, candle, piano	Basket, sled	Dustbin	Dustbin, hairbrush, sled	Candle

Swan	Ostrich, duck, chicken	Eagle, penguin, ostrich	Duck, penguin, eagle, owl, chicken	Duck, peacock	Duck, ostrich, pineapple

Tiger	Dog, cat	Mouse, squirrel, horse	Cat, eagle, piano, rabbit	Cat, dog	Elephant, turtle, squirrel

Toaster	Basket, envelope, bus	Lorry	Hairbrush, key, pliers, sled	Dustbin, helicopter, envelope	Cherry, apple, dustbin

Tomato	Apple, pineapple, orange	Cherry, apple, orange, banana	Apple, orange, cherry, banana	Apple, cherry, orange	Chicken, carrot, pineapple

Train	Bus, lorry, airplane	Airplane, helicopter, bus, lorry	Motorcycle	Bus, airplane, helicopter, motorcycle, lorry	Camel, sled

Turtle	Alligator	Elephant	Frog, stool, squirrel	Alligator	Elephant, squirrel, tiger, alligator

**Table A2:** **Lists for the three functional and the three encyclopedic sets**.

Concept	Garrard et al. functional	Cree and McRae functional	Cree and McRae edited functional	Garrard et al. encyclopedic	Cree and McRae encyclopedic	Cree and McRae edited encyclopedic
Airplane	Helicopter, sled	Train, bus	Bus, helicopter, lorry	Helicopter, piano	Helicopter	Helicopter, bus, train

Alligator	Penguin, swan, turtle	NA	Frog, ostrich, owl, peacock, penguin, turtle	Tiger	Axe, frog, scissors	Turtle, frog, peacock, penguin

Apple	Cherry, tomato, banana	Cherry	Cherry, orange, pineapple, tomato	Orange, banana, carrot, pineapple, tomato	Cherry, banana, orange, pineapple, carrot	Cherry, carrot, banana, tomato

Axe	Scissors, pliers	Scissors	Scissors, hairbrush, paintbrush	Hammer, camel, ostrich	Alligator, motorcycle, tiger	Scissors, paintbrush

Banana	Carrot, cherry, apple, orange, tomato, pineapple	Chicken, duck, rabbit, tomato	Orange, pineapple, cherry	Pineapple, orange, apple, carrot, cherry	Cherry, apple, orange, pineapple	Pineapple, orange, apple

Barrel	Dustbin, basket	NONE	Dustbin, basket	NA	NA	Dustbin, hammer, pliers, scissors

Basket	Dustbin, barrel	NONE	Dustbin, barrel	NA	NONE	Barrel, dustbin, hammer, pliers, scissors

Bike	Sled, motorcycle	Camel, horse	Camel, motorcycle, sled, horse	Motorcycle, lorry, train	NONE	Motorcycle, lorry, paintbrush, sled

Hairbrush	Paintbrush, screwdriver, pliers	NA	Paintbrush, scissors	NONE	NA	Dustbin, paintbrush

Bus	Train, lorry, motorcycle	Airplane, train	Airplane, train, helicopter	Lorry, train	NONE	Lorry, motorcycle, paintbrush

Camel	Horse, cow	Bike, horse	Elephant, bike, stool, horse	Axe, ostrich, elephant	NONE	Ostrich, elephant

Candle	Toaster, basket, stool	NONE	Lorry, stool	NONE	NONE	Paintbrush, toaster

Carrot	Banana, pineapple, orange	Tomato	Tomato, orange, pineapple	Apple, banana, cherry, pineapple, orange, tomato	Apple, tomato	Apple, tomato

Cat	Squirrel	NA	Dog	Peacock, chicken	Cow, dog, chicken	Dog, mouse, horse

Cherry	Apple, tomato, banana, orange	Apple	Apple, banana, orange, pineapple	Orange, banana, carrot, pineapple, tomato	Apple, banana, orange, pineapple	Apple

Chicken	Owl, peacock, ostrich	Duck, rabbit, banana, tomato	Duck, cow, rabbit	Peacock, cow, cat	Cow, peacock	Cow, dog, horse

Cow	Mouse, rabbit	NONE	Rabbit, chicken, duck	Chicken, rabbit, horse	Chicken, cat, dog	Chicken, dog, horse

Dog	Horse	NONE	Cat, horse, tiger	Peacock, cat, chicken	Cat, cow, chicken	Cat, cow, chicken, horse

Duck	Swan, penguin, turtle, peacock	Chicken, elephant, banana, tomato	Chicken, rabbit	Turtle, rabbit	Swan, turtle, peacock	Swan, frog, turtle

Dustbin	Basket, barrel	NA	Basket, barrel	NA	NA	Hairbrush, barrel, hammer, pliers, scissors

Eagle	Owl, swan, peacock	NA	Ostrich, owl, peacock, penguin, swan, turtle	Owl, tiger	Turtle, duck, peacock, chicken, penguin, swan	Swan, peacock, turtle, duck

Elephant	Camel, horse, cow	Tiger, duck, rabbit	Camel, horse	Ostrich	Ostrich, tiger, peacock	Tiger, camel

Envelope	Paintbrush, screwdriver, basket, sled	NONE	Lorry, stool	NA	NONE	Paintbrush

Frog	Rabbit	NA	Alligator, ostrich, owl, peacock, penguin, turtle	Duck, penguin, turtle, alligator, swan	Turtle, alligator	Turtle, alligator, penguin

Hammer	Pliers, scissors, paintbrush, screwdriver, hairbrush	Pliers, screwdriver	Pliers, screwdriver, paintbrush, scissors	Axe, camel	Pliers, screwdriver	Screwdriver, barrel, dustbin, scissors

Helicopter	Airplane, eagle	Camel, airplane	Airplane, bus, train	Airplane, motorcycle	Airplane	Airplane

Horse	Dog, camel	Bike, camel	Camel, elephant, bike	Duck, peacock, cow	Cow, peacock, chicken, swan	Cow, chicken, cat, dog

Key	Basket, dustbin	NONE	Hairbrush, paintbrush	NONE	NONE	Barrel, dustbin, hammer, pliers, scissors

Lorry	Train, bus	NA	Airplane, train	Bus, train, motorcycle	NA	Bus, motorcycle

Motorcycle	Bus, bike, train, airplane	Camel, airplane, helicopter	Bike, camel, helicopter, sled, stool	Bike, lorry, train	Axe, scissors, sled	Lorry, bike, bus, axe

Mouse	Cow, tiger, squirrel, rabbit	NONE	Squirrel, alligator, rabbit	Squirrel, dog, tiger	Squirrel	Rabbit, cat

Orange	Banana, pineapple, carrot, cherry, apple, tomato	NONE	Banana, apple, tomato, carrot, cherry	Apple, banana, cherry, pineapple	Cherry, apple, banana, pineapple	Banana, pineapple

Ostrich	Peacock	NA	Eagle, alligator, frog, swan	Camel, elephant	Peacock, elephant	Camel, turtle, peacock

Owl	Eagle, swan, chicken, peacock	NA	Eagle, alligator, frog, swan	Eagle, tiger	Squirrel	Squirrel, eagle, rabbit

Paintbrush	Screwdriver, hairbrush, pliers	NONE	Scissors, hairbrush, pliers	NONE	NA	Axe, hairbrush, piano

Peacock	Duck, swan	NA	Eagle, alligator, frog, swan	Cat, chicken, dog, horse, duck	Ostrich, chicken, penguin, swan	Turtle

Penguin	Swan, alligator, duck	NA	Eagle, alligator, frog, swan	Frog, duck, turtle, alligator, swan	Peacock, chicken, ostrich	Turtle

Piano	Stool, dustbin	NONE	Bike, lorry, motorcycle, sled, stool	Airplane	NONE	Paintbrush

Pineapple	Carrot, orange, banana, cherry	NONE	Banana, apple, tomato, carrot, cherry	Banana, orange, tomato, apple, carrot, cherry	Cherry, apple, banana, orange	Banana, orange

Pliers	Screwdriver, scissors, paintbrush, hammer	Screwdriver, hammer, horse	Hammer, screwdriver, paintbrush, scissors	NONE	Hammer, screwdriver	Screwdriver, barrel, dustbin, scissors

Rabbit	Cow, frog, tiger, squirrel	Chicken, elephant, banana, tomato	Cow, duck, alligator, chicken	Duck, cow	NONE	Squirrel, mouse, peacock

Scissors	Pliers, paintbrush, screwdriver, axe, hammer, hairbrush	Axe	Paintbrush, hairbrush, pliers	NONE	Alligator, motorcycle, tiger	Axe

Screwdriver	Paintbrush, pliers, hairbrush	Pliers, hammer	Pliers, hammer, paintbrush, scissors	NONE	Hammer, pliers	Hammer, pliers

Sled	Bike, airplane, camel	NONE	Bike, motorcycle, stool	NONE	Motorcycle	Paintbrush, bike

Squirrel	Cat, mouse, rabbit	NA	Mouse, alligator, rabbit	Turtle, rabbit	Owl, mouse	Rabbit, owl

Stool	Piano, toaster, basket, candle, dustbin	NONE	Camel	NA	None	Paintbrush, toaster

Swan	Penguin, duck, alligator	NA	Eagle, ostrich, owl, peacock, penguin, turtle	Alligator	Duck, peacock	Duck, eagle, peacock

Tiger	Mouse, rabbit, cat, squirrel	Elephant	Eagle, alligator	Alligator, eagle, owl	Elephant, axe, scissors, ostrich	Elephant

Toaster	Candle, basket, stool, dustbin	NONE	Lorry, stool	NONE	NONE	Paintbrush, stool

Tomato	Cherry, apple, banana	Chicken, duck, rabbit, banana	Carrot, orange, pineapple, apple	Pineapple, apple, banana, carrot, cherry	Carrot	Carrot, apple, paintbrush

Train	Bus, lorry, motorcycle	Airplane, bus	Bus, helicopter, lorry	Lorry, bus, motorcycle	NONE	Bus, paintbrush

Turtle	Duck, alligator, penguin	NA	Eagle, alligator, frog, swan	Duck, squirrel, ostrich	Frog	Peacock, alligator, penguin, ostrich
